# Swallowing-related quality of life after free flap surgery due to cancer of the head and neck

**DOI:** 10.1007/s00405-018-05264-w

**Published:** 2018-12-28

**Authors:** Sanna Lahtinen, Petri Koivunen, Tero Ala-Kokko, Outi Kaarela, Päivi Laurila, Janne H. Liisanantti

**Affiliations:** 1 Research Group of Surgery, Anesthesia and Intensive Care, Department of Anesthesiology, Medical Research Center, Oulu University Hospital, University of Oulu, P.O. Box 21, 90029 Oulu, Finland; 2 PEDEGO Research Unit, Department of Otorhinolaryngology and Head and Neck, Medical Research Center, Oulu University Hospital, University of Oulu, Oulu, Finland; 3 Research Group of Surgery, Anesthesia and Intensive Care, Department of Surgery, Medical Research Center, Oulu University Hospital, University of Oulu, Oulu, Finland; 4 Division of Operative Care and Medical Research Center Oulu, Oulu University Hospital, University of Oulu, Oulu, Finland

**Keywords:** Cancer of the head and neck, Free flap surgery, Dysphagia, Quality of life

## Abstract

**Purpose:**

Treatment of head and neck cancers (HNC) often leads to impairment in speech and swallowing functions. This study evaluated swallowing problems and the impact of complications on swallowing-related QOL after free flap surgery for HNC.

**Methods:**

Swallowing-related QOL was assessed using MDADI and SWAL questionnaires.

**Results:**

Of 45 assessed patients, 25 (45.5%) had at least one postoperative complication. Patients reported less than < 86 points in 8/9 SWAL-QOL domains. The SWAL-QL total score or MDADI composite scores were not related to surgical complications. Those with medical complications had lower scores in SWAL-QOL domains of mental health (82.8 (21.8) vs 65.5 (24.2), *p* = 0.024) and sleep (77.6 (23.0) vs 52.3 (24.3), *p* = 0.003).

**Conclusions:**

In conclusion, swallowing related QOL is significantly impaired after 2 years of the tumor resection and free flap reconstruction for cancer of the head and neck, when using the cut-off value of 86 points in SWAL-QOL assessment tool. Surgical complications did not have an impact on swallowing-related QOL but medical complications were related to impairment in general QOL-related domains.

## Introduction

Cancer of the head and neck and its surgical and oncological treatment include high risk of complications and also the reported 5-year mortality is high [1]. Moreover, the operative care often leads to impairment in every-day life functions such as swallowing, speech, and physiological airway. Despite of the development in the treatments, dysphagia is still a common problem in this patient group and may have a significant impact on quality of life (QOL) [2–4].

The few available studies report the swallowing problems as a part of disease-specific QOL or focus on evaluating the function and physiology of swallowing, but not its impact on QOL [5, 6]. Accordingly, various methods have been used in studies reporting the impact of free flap reconstruction on swallowing problems and the results of these studies are partly contradictory [6–9]. Despite the reasonably high rate of postoperative complications, their role in swallowing-related QOL is also unclear. It could be hypothesized that surgical complications resulting in prolonged recovery and impaired postoperative function could have a major impact on the swallowing-related QOL. Therefore, we determined to evaluate the impact of postoperative complications on swallowing-related QOL using swallowing-specific QOL questionnaires in head and neck cancer patients operated with free flap reconstruction.

## Methods

This study was conducted in Oulu University Hospital and the study protocol was approved by the hospital administration (239/2016) and local ethics committee (The Regional Ethics Committee of the Northern Ostrobothnia Hospital 95/2016). The present study is a substudy of our previously published paper examining general QOL in relation with postoperative complications in patients with free flap reconstruction due to cancer of the head and neck [1].

### Study population

Between 31st of January 2013 and 31st of December 2016, there were a total of 90 patients that underwent free flap surgery due to cancer of the head and neck. Of these, 53 were still alive at the time of assessment. We assumed that swallowing-related quality of life is impaired in those with intra-oral or laryngeal tumour. Of the total 53 patients, five had maxilla tumour, two had melanoma, and one patient had lymphoma. These patients were excluded, resulting in 45 patients, whose reconstruction affected the anatomy and function of oral cavity or larynx.

### Procedure

The swallowing-related QOL of patients was assessed by interview using validated tools (SWAL-QOL and MDADI) [10, 11]. To assess general QOL, RAND-36 tool was used [12]. Mean time from operation to interview was 111.2 weeks (SD 48.6).

### Swallowing quality-of-life questionnaire (SWAL-QOL)

SWAL-QOL consists of 44 questions assessing ten QOL domains: food selection, burden, mental health, social functioning, fear, eating duration, eating desire, communication, sleep, and fatigue. Sleep and fatigue contribute to general QOL, whereas the other domains are contributors to dysphagia-specific QOL. For analyzing the results, each scale is constructed using Likert`s method, which equally weighs each item and sums them into an overall scale sore. All scales are transformed to a 0–100 metric, 100 indicating the most favorable state and 0 the least favorable, and scores in between representing the percentage of the total possible score achieved. SWAL-QOL is developed to assess the impact of oropharyngeal dysphagia in neurologic diseases, but has also been used in other conditions causing swallowing problems, i.e., head and neck cancer [10, 13, 14]. The total SWAL-QOL score includes 23 items from seven domains (communication, sleep and fatigue not included) [10]. A cut-off value of 86 is used to determine significant impairment of swallowing-related QOL [15].

### M.D. Anderson dysphagia inventory (MDADI)

MDADI is validated and a reliable self-administered questionnaire designed specifically to measure the impact of swallowing problems on QOL of patients with cancer of the head and neck. It consists of four subdomains: global (one question), emotional (six questions), functional (five questions), and physical (eight questions). The global question illustrates patient`s overall QOL and the other domains indicate the impact of dysphagia on patient`s affective response, daily activities, and self-perception. Each item is rated from one to five on a five-point scale. The global question is scored individually, while a sum of the other scores and a mean score are calculated. This mean score is multiplied by 20 to obtain a MDADI total score with a range of 20–100; the higher the score, the better the QOL [11, 16, 17].

### The RAND 36-item short-form health survey (RAND-36)

The RAND-36 consists of 36 questions in eight health domains: physical functioning (ten questions), role limitations due to physical health problems (four questions), pain (two questions), general health (five questions), energy/fatigue (four questions), social functioning (two questions), role limitations due to emotional problems (three questions), emotional well-being (five questions), and one question concerning change of health during the last year. The answers for each domain are transformed a score between 0 (worst) and 100 (best). Physical and mental health summary scores can be derived from the eight domains. The RAND-36 is widely used to compare the health- related QOL of general and specific populations. It is also often used for testing validity and reliability of new QOL instruments. The RAND-36 was introduced in 1990's and has been used worldwide for decades [3, 12].

If a patient had at least one RAND-36 domain score less than − 2SD below age-adjusted population values, QOL was considered poor.

### Data extraction

Patient demographics were retrieved from the medical records. The recorded complications included surgical and medical complications. The classification of complications is the same used in our previously published study focusing the postoperative complications in this same patient cohort, including all the patients who were operated due to cancer of the head and neck with free flap repair in Oulu University Hospital during 2008–2015 [1].

### Statistical analysis

Continuous variables are presented as mean and standard deviation (SD) and categorical variables as numbers and percentages (%). Non-parametric Mann–Whitney test was used to compare continuous variables and Pearson’s Chi-square or Fisher’s exact test for categorical variables. A *p* value below 0.05 was considered statistically significant. Statistical analysis was performed using SPSS software.

## Results

A total of 45 patients were included into the analysis, which of 25 (45.5%) had at least one recorded complication. Of those with recorded complications, 20 (80%) had surgical complications and 11 (44%) had medical complications. The frequencies of the recorded complications are presented in Table 1. Ten (40%) of the patients with recorded complications had been reconstructed with ALT (anterolateral thigh) as free flap (Table 2). Eight (32%) of the patients with recorded complications had at least one RAND-36 domain below − 2SD of age-adjusted general values in contrast to one in uncomplicated patients (*p* = 0.024).

**Table 1:** Frequencies of the recorded complications

Recorded complication	Number of recorded complications
Surgical complications	
Surgical site infection	5
Postoperative hemorrage	11
Need for reoperation/exploration	14
Partial flap failure	2
Flap failure	1
Medical complications	
Pneumonia	8
Sepsis	2
Pulmonary ödema	2
Acute myocardial infarctation	1
Acute kidney injury	1
Deep venous thrombosis, pulmonary embolism, stroke	0

**Table 2:** Demographic data of 45 patients

	No complication *n* = 20	Complication *n* = 25	Missing data	*p*-value
Gender (m)	5 (25.0)	13 (52.0)	0/0	0.066
Age (years)	67.4 (8.6)	62.1 (8.5)	0/0	0.076
Free flap			0/0	
RFA	12 (60.0)	8 (32.0)		
ALT	0 (0.0)	10 (40.0)		0.032
Fibula	4 (20.0)	4 (16.0)		
Lateral arm	3 (15.0)	2 (8.0)		
Others	1 (5.0)	1 (4.0)		
Tumor			0/0	
Oral cavity/tongue	9 (45.0)	14 (56.0)		
Mandibula	6 (30.0)	5 (20.0)		
Larynx/pharynx	0 (0.0)	2 (8.0)		0.074
Palatinal	4 (20.0)	0 (0.0)		
Buccal mucosa	1 (5.0)	4 (16.0)		
Tumor stage			1/1	
T1–2	11 (55.0)	13 (54.2)		0.956
T3–4	9 (45.0)	11 (45.8)		
≥ 1 RAND-36 domain below − 2SD of age adjusted general population	1 (5.0)	8 (32.0)	0/0	0.024

*RFA* radialis forearm, *ALT* anterolateral thigh

### Swallowing-related QOL assessed using SWAL-QOL and MDADI

There were no differences in SWAL-QOL domains except sleep or any MDADI domains between patients with or without postoperative complications. All SWAL-QOL domains except fear of eating were below 86 (Tables 3, 4). There were no differences in swallowing-related QOL between patients with or without surgical complications when assessed with SWAL-QOL and MDADI (data not shown). Those with medical complications had lower scores in SWAL-QOL domains of mental health (82.8 (21.8) vs 65.5 (24.2), *p* = 0.024) and sleep (77.6 (23.0) vs 52.3 (24.3), *p* = 0.003). There were no differences in other domains of SWAL-QOL or MDADI.

**Table 3:** SWAL-QOL scores of patients with and without complication

SWAL-QOL domain	No complication *n* = 20 (mean, SD)	Complication *n* = 25 (mean, SD)	*p*-value
Burden	62.5 (41.2)	56.5 (35.4)	0.471
Eating duration	38.1 (26.4)	29.5 (30.6)	0.183
Eating desire	80.4 (27.1)	69.0 (29.6)	0.208
Symptom frequency	76.4 (16.0)	72.9 (17.8)	0.437
Food selection	77.5 (25.2)	71.5 (28.1)	0.549
Communication	72.5 (25.2)	66.5 (28.1)	0.493
Fear of eating	93.4 (15.0)	94.7 (10.2)	0.944
Mental health	80.3 (23.3)	77.2 (23.9)	0.552
Social functioning	76.5 (30.4)	73.6 (29.3)	0.887
Fatigue	81.7 (21.0)	73.7 (20.8)	0.133
Sleep	79.4 (25.4)	65.0 (24.2)	0.024
SWAL-QOL total	76.4 (18.4)	72.3 (18.3)	0.411

**Table 4:** MDADI scores of patients with and without complication

MDADI subscale	No complication *n* = 20 (mean, SD)	Complication *n* = 25 (mean, SD)	*p*-value
Emotional	69.3 (17.9)	68.1 (15.1)	0.909
Functional	77.2 (19.6)	72.5 (20.1)	0.429
Physical	78.5 (16.0)	75.1 (19.1)	0.531
Global score	75.0 (32.4)	64.0 (30.6)	0.244
Composite score	75.3 (16.5)	72.5 (17.0)	0.555

Patients with significantly impaired swallowing-related QOL (SWAL-QOL cutoff < 86), reported worse scores in a total of 3 of 8 RAND-36 domains [vitality 67.4 (15.1) vs 81.9 (12.8), *p* = 0.005, mental health 78.6 (12.6) vs 87.5 (9.9), *p* = 0.017 and social functioning 79.3 (18.1) vs 89.1 (21.8), *p* = 0.023] (Table 5).

**Table 5:** Patients categorized using SWAL-QOL cut-off value of 86 as indicator for impaired swallowing-related QOL

	SWAL-QOL total < 86 *n* = 29	SWAL-QOL total ≥ 86 *n* = 16	*p*-value
Gender (m)	15 (51.7)	3 (18.8)	0.031
Age (years)	64.5 (8.9)	64.4 (9.1)	0.233
BMI	24.3 (5.6)	25.5 (5.7)	0.487
Time from operation (w)	100.4 (45.7)	130.0 (49.5)	0.054
Preoperative albumin	42.8 (3.6)	44.3 (2.1)	0.232
Tumor-stage > 2*	12/28 (42.9)	8/16 (50.0)	0.647
Hospital LOS (d)	14.4 (7.5)	7.9 (1.3)	0.005
Tumor			
Oral cavity/tongue	17 (58.6)	6 (35.3)	
Mandibula	6 (20.7)	5 (31.3)	
Larynx/pharynx	2 (6.9)	0 (0.0)	0.387
Palatinal	2 (6.9)	2 (12.5)	
Buccal mucosa	2 (6.9)	3 (18.8)	
Free flap			
RFA	13 (44.8)	7 (43.8)	
ALT	8 (27.6)	2 (12.5)	
Fibula	5 (17.2)	3 (18.8)	0.634
Lateral arm	2 (6.9)	3 (18.8)	
Others	1 (3.4)	1 (6.3)	
Surgical complication	15 (51.7)	5 (31.3)	0.186
Medical complication	10 (34.5)	1 (6.3)	0.035**
Any complication	19 (65.5)	6 (37.5)	0.070
RAND-36 domain			
Physical functioning	73.6 (29.8)	85.6 (24.9)	0.222
Role physical	65.9 (35.0)	75.8 (33.4)	0.296
Role emotional	74.7 (32.9)	83.3 (36.5)	0.187
Vitality	67.4 (15.1)	81.9 (12.8)	0.005
Mental health	78.6 (12.6)	87.5 (9.9)	0.017
Social functioning	79.3 (18.1)	89.1 (21.8)	0.023
Pain	72.6 (24.3)	82.7 (20.0)	0.187
General health	51.7 (20.4)	60.3 (21.8)	0.171
1 ≥ RAND-36			
Domain below 2SD age adjusted general values	7 (24.1)	2 (12.5)	0.350
MDADI subscale			
Emotional	60.9 (14.5)	82.7 (7.3)	< 0.001
Functional	65.2 (18.1)	91.5 (8.0)	< 0.001
Physical	67.9 (15.6)	91.9 (8.0)	< 0.001
Global score	55.2 (29.6)	93.8 (15.9)	< 0.001
Composite score	65.1 (14.4)	88.9 (6.0)	< 0.001

*BMI* body mass index, *LOS* length of stay, *RFA* radialis forearm, *ALT* anterolateral thigh

*One missing tumour stageing in both groups

**Fisher's exact test used for comparing categorical variables

## Discussion

The main finding of our study is that swallowing-related QOL is still impaired after 2 year recovery from free flap surgery for the cancer of head and neck; more than 60% of the patients had significantly impaired swallowing-related QOL when using the SWAL-QOL cut-off value 86. Interestingly, we found only minor relation between complications and swallowing-related QOL; the scores for SWAL-QOL domains “sleep” and “mental health” were lower in those with medical complications. These domains are likely to be less swallowing-related instead of general domains. Finally, according to our results, impairment in swallowing-related QOL is not easily detected using RAND-36 tool.

There are no previous studies focusing the impact of complications on the swallowing-related QOL. Previous studies are mainly focusing on swallowing function at 6 and 12 months [5] have used general QOL tools [7] or have assessed swallowing by diet [8]. Interestingly, in our cohort, the SWAL-QOL scores were equally below 86 despite surgical complications were absent or not. This could be explained by the recovering period; patients were interviewed more than year from the operation. According to previous studies, 1 year is a time period which is needed to achieve a stabile state or a return to pre-treatment level in head and neck specific symptoms after operation [2, 18, 19]. When interviewed more than 1 year from the operation, the patients with surgical complications might have achieved sufficient recovery, even when taking into account that in the present study, the recorded surgical complications can be considered major; for instance, 70% of those with surgical complication required exploration or reoperation. In addition, the large reconstruction of the oral cavity or laryngeal area has a deteriorated effect on swallowing function itself which can hide the impact of surgical complications on swallowing-related QOL [5].

In our patients, the medical complications had an impact on swallowing-related QOL in SWAL-QOL domains of sleep and mental health. These domains present rather general QOL instead of disease-specific QOL or functional problems in daily life. In addition, nearly, all the patients with medical complications reported SWAL-QOL less than 86. These results are in line with our previous findings concerning general QOL in this patient group and highlight the important role of medical complications on compromising the recovery process [1].

### Clinical impact

According to our results, recognition of risk population for swallowing problems is demanding. It is previously reported that patients with tongue, soft palate, and laryngeal reconstructions are in risk of impaired swallowing function postoperatively, but we were not able to show any other patient-related risk factors [4, 5, 20]. We found a relation between RAND-36 values and impaired swallowing-related QOL life in 3 of 7 RAND-36 domains, which were more or less linked to psycho-social aspects when assessed using SWAL-QOL cut off value 86. Swallowing-related QOL does not automatically appear in general QOL measurements. Therefore, patients should be screened for swallowing-related problems in postoperative phase and logopedists and physiotherapists as well as nutritionists should be invited to the rehabilitation process. Moreover, prolonged follow-up should be established, since the data concerning spontaneous recovery are lacking. In our previous study, low preoperative albumin level and low BMI were indicative for 5-year mortality after free flap surgery for cancer of the head and neck [21]. These are often caused by malnutrition. Swallowing-related problems can cause further malnutrition postoperatively and by that impair the recovery process and also may have an impact on mortality.

### Limitations

This study has its limitations. First, we were able to include a limited number of patients and a limited number of different of tumours. Second, the retrieved data concerning operative care, patient demographics, and postoperative complications were retrospective. Third, it would have been interesting if we could have been able to include objective measurement of swallowing function, i.e., naso-fiberoscopy in the present study. Fourth, we were not able to include assessment of nutritional status, such as BMI or albumin levels to the analysis, which could have had been compromised in those with deteriorated swallowing-related QOL. We also did not analyse the impact of other treatment modalities including oncological treatment on swallowing-related QOL.

## Conclusions

In conclusion, swallowing-related QOL is significantly impaired after 2 years of the tumor resection and free flap reconstruction for cancer of the head and neck when using the cut-off value of 86 points in SWAL-QOL assessment tool. Surgical complications did not lead to impairment of swallowing-related QOL and patients with medical complications reported more often impairment in general QOL-related domains such as sleep and mental health.
